# Partial abdominal carbon-ion FLASH irradiation spares lethality compared to conventional irradiation: impact of LET and dose rate

**DOI:** 10.1186/s13014-025-02765-x

**Published:** 2025-11-28

**Authors:** Wenteng Cao, Yukari Yoshida, Hiromu Suda, Shunsuke Inagaki, Naoto Urabe, Masao Nakao, Ken Yusa, Xiangdi Meng, Mutsumi Tashiro, Akihisa Takahashi, Tatsuya Ohno

**Affiliations:** 1 Department of Radiation Oncology, Gunma University Graduate School of Medicine, Maebashi, Japan; 2 Gunma University Heavy Ion Medical Center, Maebashi, Japan

**Keywords:** FLASH, Carbon-ions, Mouse intestine, Dose rate, Linear energy transfer (LET)

## Abstract

**Background:**

Ultra-high dose rate (FLASH) therapy has attracted attention for its potential to effectively target tumors while preserving healthy tissues. Although the biological effects of FLASH therapy have been well-documented with electrons, photons, and protons, data regarding carbon-ions are limited. This study aimed to investigate the biological effects of carbon-ions on the intestines of mice to determine whether carbon-ions exhibit the FLASH effect. Simultaneously, we aimed to identify the parameters influencing its occurrence.

**Materials and methods:**

Partial abdominal irradiation was performed on C3H/He mice using 290 MeV/u FLASH and conventional dose rate (CONV) carbon-ions under both low and high linear energy transfer (LET). The evaluation included the 30-day survival rate, body weight changes, and stool pellet alterations within 14 days after irradiation. Furthermore, we assessed the number of regenerating crypts 3.5 days after irradiation.

**Results:**

Under low-LET, FLASH and CONV irradiation exhibited comparable sparing effects on normal intestinal tissues. However, FLASH significantly improved the survival rate of mice under high-LET, when the dose rate exceeded 100 Gy/s. The number of regenerating crypts 3.5 days after irradiation was 1.5 times higher in the FLASH group than in the CONV group.

**Conclusions:**

The results indicate that carbon-ion FLASH exhibits the FLASH effect at high-LET with dose rate and LET identified as critical parameters influencing the FLASH effect.

**Supplementary Information:**

The online version contains supplementary material available at 10.1186/s13014-025-02765-x.

## Introduction

Carbon-ion therapy leverages the unique physical properties of carbon-ions to enhance cancer treatment precision and effectiveness. This precise localization of energy deposition enables the delivery of higher radiation doses to tumors while minimizing irradiation to surrounding normal tissues. Furthermore, carbon-ions show a 2–3-fold greater antitumor effect than photons [1]. However, even with these advanced radiotherapy modalities, some tumors remain incurable because of insufficient dose delivery (e.g., highly radioresistant tumors or tumors surrounded by radiosensitive organs), highlighting the need to establish methods that further expand the therapeutic window of radiotherapy.

FLASH radiotherapy is an innovative approach that delivers ultra-high dose rates exceeding 40 Gy/s, aiming to widen the therapeutic window by minimizing damage to normal tissue while maintaining tumor control. The resulting biological phenomenon, known as the FLASH effect, has been primarily demonstrated with various types of radiation, including electrons [2–4], photons [5–7], and protons [8–11] in mice [12, 13], zebrafish embryos [14, 15], mini pigs, and cats [16] with multiple normal tissues, including the lung [2, 17], intestine [3, 5, 18, 19], head [6, 20, 21], and skin [11, 12, 22]. However, biological data on the effects of FLASH radiotherapy using carbon-ions are limited, and only a few in vivo [23, 24] and in vitro [25–27] studies have been conducted to date. Currently, a study showed that although the concept of FLASH defines dose rates exceeding 40 Gy/s, achieving the full effect requires a dose rate equal to or even higher than 100 Gy/s, and several findings have supported this view [28–31]. Therefore, the dose rate is an important parameter influencing the FLASH effect. However, this view has not yet been confirmed in the context of carbon-ion FLASH irradiation, and the relationship between dose rate and the FLASH effect in carbon-ion therapy remains poorly understood.

When performing FLASH irradiation with carbon-ions in clinical settings, it is important to clarify the FLASH effects for each linear energy transfer (LET). In carbon-ion therapy, a mixture of carbon-ions with different energies is used near the tumor to obtain a spread-out Bragg peak (SOBP) and variable LET [32]. This study aimed to investigate the biological effects of carbon-ion FLASH irradiation on the mouse intestine using different dose rates under both low- and high-LET conditions. The mouse intestine is an excellent model for studying radiotherapy for several reasons: it is highly sensitive to radiation due to the rapid turnover of its epithelial cells [33], and the physiological and histological structure of the mouse intestine is similar to that of humans [34]. Furthermore, it provides clear, quantifiable endpoints for evaluating radiation effects and is relevant for understanding gastrointestinal toxicity [33]—a significant limiting factor in radiotherapy dose escalation. This study aimed to reveal the potential benefits of carbon-ion FLASH therapy through a systematic analysis of intestinal function and histology, laying the groundwork for further exploration of its underlying mechanisms. Furthermore, this study aimed to identify the parameters influencing the FLASH effect.

## Materials and methods

### Mouse model

Female C3H/He mice (8–11 weeks old) were purchased from SLC Co., Ltd (Shizuoka, Japan) and allowed to acclimate for 1 week before irradiation. Animals were housed under specific pathogen-free conditions with a 12-h light/dark cycle and *ad libitum* access to sterile food and water. Mice were randomly assigned to three groups: nonirradiated control, conventional dose rate (CONV) irradiation, and FLASH irradiation. A total of 164 mice were included in the final analysis. All follow-up assessments were conducted in a blinded fashion with respect to treatment allocation. All experiments complied with the ARRIVE guidelines (Animal Research: Reporting of In Vivo Experiments). All animal procedures were performed according to the Recommendations for Handling of Laboratory Animals for Biomedical Research and were approved by the Animal Care and Experimentation Committee of Gunma University (No. 23 − 005; Application date: 30 March 2023).

### Irradiation setups

**Fig. 1:**
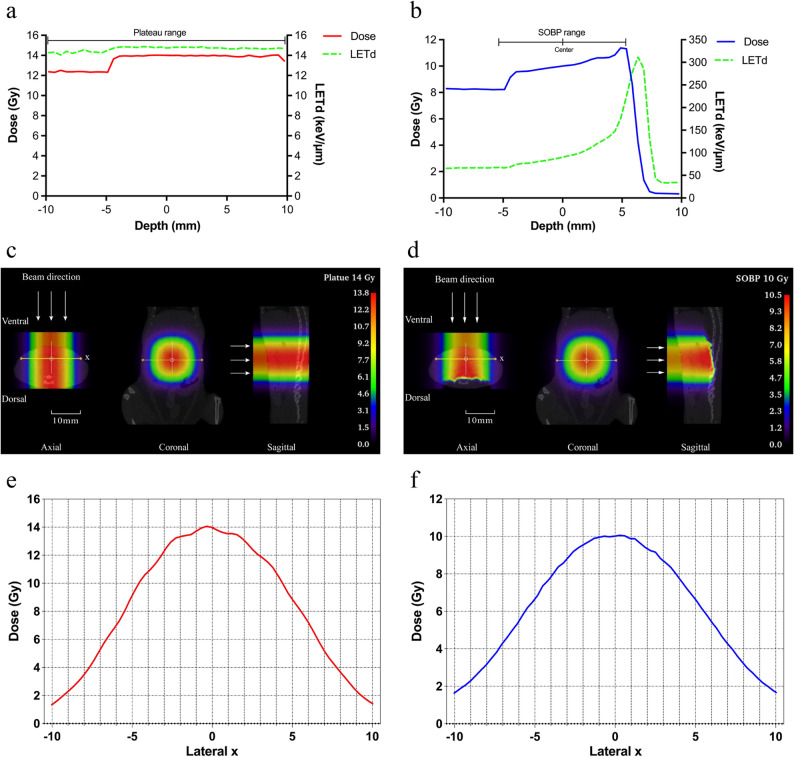
Experimental setup for FLASH and CONV irradiation. Depth-dose and depth-LETd curves of plateau range at 14 Gy (**a**) and the SOBP range at 10 Gy (**b**). Dose profiles (red/blue solid lines, left Y-axis) and dose-averaged linear energy transfer (LETd, green dashed line, right Y-axis) were obtained from simulations performed using PHITS version 3.32. The depth corresponds to the beam direction shown in (**c**) and (**d**), along the axial plane passing through the center of the irradiation field. In the simulation, particle transport was based on CT derived tissue equivalent materials, and LETd values were calculated for water with a density of 1 g/cm^3^. (**c**, **d**) Axial, coronal, and sagittal CT images showing spatial dose distributions simulated using 3D Slicer in the abdominal region under 14 Gy low-LET (**c**) and 10 Gy high-LET (**d**) carbon-ion irradiation. The carbon-ions were delivered perpendicularly from the ventral (abdominal) side to the dorsal (back) side, as the arrows in the figures. View orientations (axial, coronal, sagittal) and anatomical directions (ventral, dorsal) are labeled. The color bar represents dose levels in gray (Gy). The yellow lines in the axial and coronal views mark the locations where dose profiles were extracted. The intersection of the yellow horizontal line and the white vertical dashed line indicates the center point. (**e**, **f**) Lateral dose profiles corresponding to 14 Gy low-LET (**e**) and 10 Gy high-LET (**f**) carbon-ion irradiation, measured along the yellow lines defining the lateral x-axis shown in (**c**) and (**d**), respectively

All irradiation procedures were performed using a scanning vertical beam port at Gunma University Heavy Ion Medical Center. The setup and dosimetry parameters for both FLASH and CONV irradiation in this study were consistent with those employed in our center’s previous research [27]. Using the design data of a ridge filter that produces a 10 mm-wide SOBP, the dose-averaged linear energy transfer (LETd) of a 290 MeV/u carbon-ions was calculated. The LETd ranges from 60 keV/µm at the proximal side to 175 keV/µm at the distal side within the SOBP. The LETd at the center of the SOBP is 90 keV/µm (high-LET), whereas the LETd at the plateau (entrance) region of the SOBP is 15 keV/µm (low-LET). Based on previous studies at our center on intestinal crypt survival [35], doses of 14 and 16 Gy in the low-LET region and 8 and 10 Gy in the high-LET region were determined. The depth–dose and depth–LETd curves for the SOBP 10 Gy and plateau 14 Gy beams are shown in (Fig. 1a and b), illustrating the characteristic variation in LETd with irradiation depth. In the FLASH group, the dose rate ranged from 52.8 to 158.2 Gy/s, with average dose rates of approximately 102 and 110 Gy/s in the low- and high-LET groups, respectively, meeting the definition of FLASH irradiation (>40 Gy/s). In contrast, the CONV group had dose rates of 0.051–0.054 Gy/s. To investigate the relationship between dose rate and the effect of carbon-ion FLASH irradiation in greater detail, the FLASH group was subdivided into two groups at the SOBP center with 10 Gy based on dose rates either below or exceeding 100 Gy/s, with average dose rates of 66 and 154 Gy/s, respectively (Table 1). All mice received intraperitoneal injections of ketamine and xylazine for anesthesia before irradiation. The control group received the same doses of anesthesia but was not irradiated. Mice were immobilized on a custom-designed acrylic plate, with their limbs taped to prevent movement during irradiation. A localized abdominal area was irradiated using isocentric laser targeting (Suppl. Figure 1). Before irradiation, computed tomography scans were performed on mice of the same age and body weight as the experimental group to ensure precise positioning. Using external markers, the irradiation center was set 2.5 cm cranially along the abdominal midline from the external urethral orifice, enabling a 10 × 10 mm partial abdominal irradiation field. This field size was approximately 70% of the whole abdominal irradiation, and this approach ensured consistency and repeatability across all irradiation procedures. After irradiation, the dose and dose distribution were simulated and analyzed. The simulated dose distributions for low-LET 14 Gy (Fig. 1c and e) and high-LET 10 Gy (Fig. 1d and f) showed that radiation doses were primarily concentrated in the targeted abdominal irradiation area, showing a distinct localized high-dose region (red zone). The irradiation field was precisely confined to the small intestine in the axial, coronal, and sagittal views, with uniform dose distribution and well-defined boundaries.

**Table 1:** Comparison of irradiation parameters across different modalities and LET conditions

LETd (keV/µm)	Total Dose	Modality	Maximum Dose Rate (Gy/s)	Minimal Dose Rate (Gy/s)	Mean Dose Rate (Gy/s)
15	14 Gy	CONV	0.054	0.052	0.053
		FLASH	153.3	52.8	102.3
	16 Gy	CONV	0.054	0.052	0.053
		FLASH	153.3	52.8	102.3
90	8 Gy	CONV	0.052	0.051	0.051
		FLASH	158.2	64.5	110.3
	10 Gy	CONV	0.052	0.051	0.051
		FLASH	158.2	64.5	110.3
90	10 Gy	CONV	0.052	0.051	0.051
		FLASH < 100 Gy/s	72.1	64.5	66.4
		FLASH >100 Gy/s	158.2	150.2	154.2

Abbreviations: LETd: dose-averaged Linear energy transfer; CONV: Conventional dose rate; FLASH: ultra-high dose rate

### Survival and intestinal function assay

Mice were group-housed before irradiation and individually housed afterward. Survival was monitored daily for 30 days after irradiation. The mice were observed without a predetermined time point for euthanasia, and their survival status was recorded daily until 30 days after irradiation to capture the full range of radiation-induced lethality.

Intestinal function was assessed by recording daily body weight and quantifying the number of stool pellets formed at predefined time points up to 14 days after irradiation. A nonirradiated control group was maintained under identical housing and monitoring conditions as a baseline reference. Diarrhea and reduced fecal output were considered key indicators of gastrointestinal radiation toxicity.

### Histological and regenerating crypt analysis

The intestinal tissues of the mice were collected 3.5 days (84 h) after irradiation. The mice were anesthetized using an intraperitoneal injection of ketamine and xylazine, the same as for irradiation. The intestinal tissues were sampled from the proximal jejunum, approximately 3–5 cm distal to the stomach, corresponding to regions consistently located within the irradiation field. The obtained tissues were fixed by immersion in 10% neutral buffered formalin, embedded in paraffin, sectioned at 4 μm, and stained with hematoxylin and eosin. Three transverse sections of the intestinal tissues were analyzed per mouse to count the number of regenerating crypts. Sections were included in the analysis if they met the following criteria: (1) a complete jejunal circumference was present, and (2) the mucosa was oriented perpendicular to the long axis of the intestine. Crypts were considered regenerating if they contained >10 basophilic crypt epithelial cells [5, 18, 31].

### Statistical analyses

Data are presented as means ± standard deviations, and statistical analyses were performed using Prism v10 (GraphPad Software, CA, USA). Survival rate outcomes were summarized using Kaplan–Meier curves and analyzed using the log-rank test. Comparisons between two groups were performed using an unpaired Student’s *t*-test, whereas comparisons among multiple groups were performed using an ordinary one-way analysis of variance, followed by Tukey’s *post hoc* multiple comparisons test. *P*-values < 0.05 were used to denote statistical significance.

## Results

### Comparison of FLASH and CONV irradiated mice under low-LET (15 keV/µm)

**Fig. 2:**
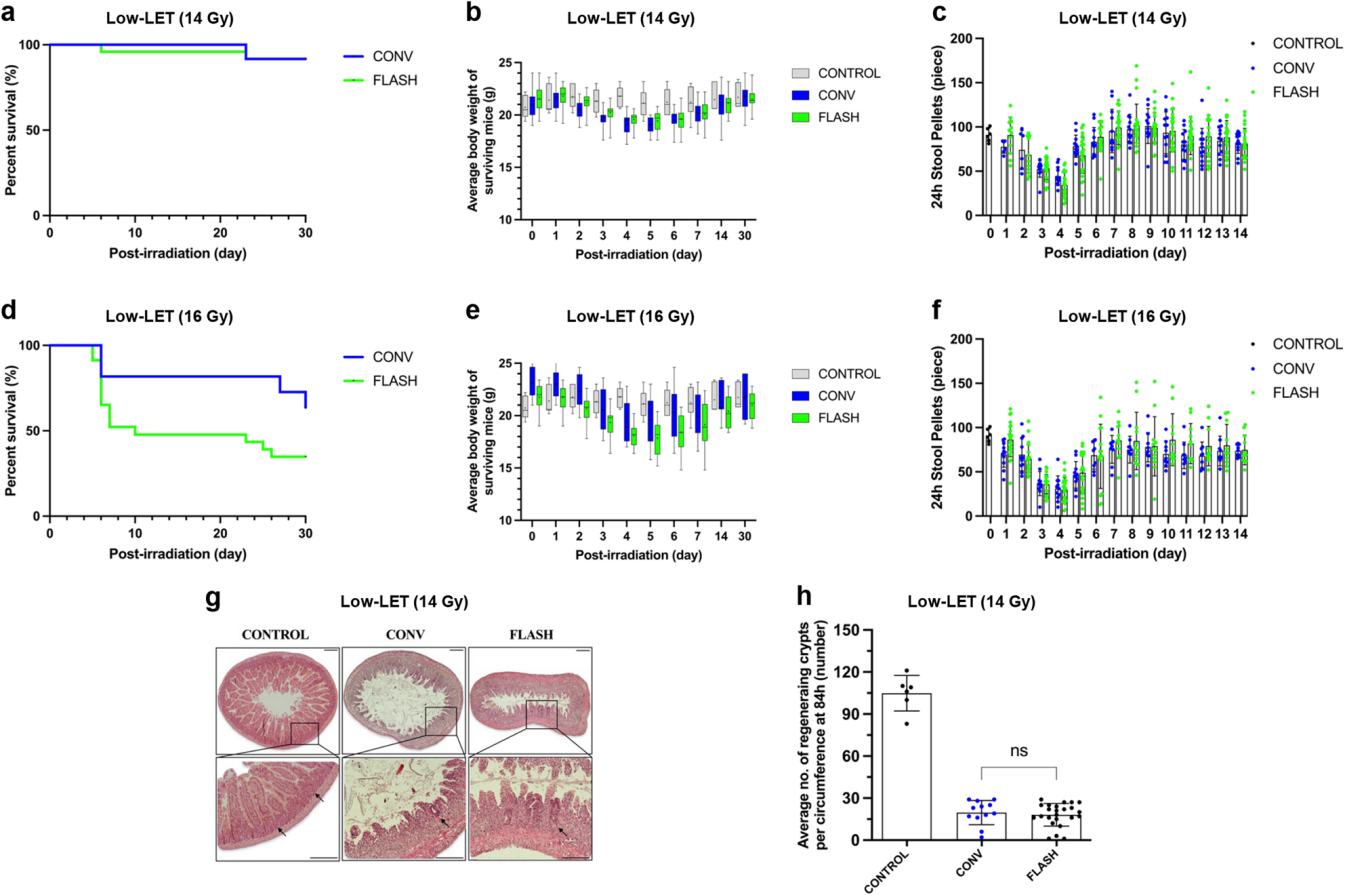
Comparison of FLASH and CONV irradiations under low-LET (15 keV/µm) conditions. (**a**) Survival rate curve of mice that received 14 Gy FLASH or CONV abdominal irradiation in 30 days. (**b**) Daily body weight changes of surviving mice 30 days after irradiation. Each point represents the mean body weight (g) ± standard deviation recorded for all mice in each group at specific time points. (**c**) Quantification of the number of formed stool pellets excreted by unirradiated control mice and mice irradiated with 14 Gy over 24 h at specified time points. (**a**–**c**) *n* = 6 in the control group, *n* = 12 in the CONV group, and *n* = 24 in the FLASH group. (**d**) Survival rate curve of mice that received 16 Gy FLASH or CONV abdominal irradiation in 30 days. (**e**) The daily body weight changes of surviving mice 30 days after irradiation. Each point represents the mean body weight (g) ± standard deviation recorded for all mice in each group at specific time points. (**f**) Quantification of the number of formed stool pellets excreted by unirradiated control mice and mice irradiated with 16 Gy over 24 h at specified time points. (**g**) Representative hematoxylin and eosin (H&E)-stained cross-sections of the mouse small intestine at 84 h after irradiation in the control, CONV, and FLASH groups after irradiation. The upper panels show low-magnification views of intestinal morphology; the lower panels present magnified images of the boxed regions. Arrows indicate regenerating intestinal crypts observed 84 h after irradiation. scale bar 100 μm. (**h**) Quantification of the average number of regenerating crypts after irradiation. (**d**–**f**) *n* = 6 in the control group, *n* = 11 in the CONV group, and *n* = 23 in the FLASH group. (**g**–**h**) *n* = 4 in the CONV and FLASH groups

The FLASH and CONV groups for 14 Gy in low-LET had the same survival rates of 92% at 30 days after irradiation, respectively (Fig. 2a). The body weight decreased from day 2 and began to recover on day 6 after irradiation, and the maximum average body weight loss occurred on day 5 after irradiation, reaching 10% in the FLASH group and 9% in the conventional group. Most irradiated mice recovered to their original body weight within 30 days after irradiation (Fig. 2b). Irradiated mice exhibited marked changes in stool characteristics, including decreased quantity, irregular shape, and increased moisture. The number of pellets began to decrease on day 2 after irradiation, reached a minimum on day 4, and recovered to normal by day 7 in two groups. However, no noticeable differences were observed between the two irradiated groups (Fig. 2c).

At 16 Gy under low-LET conditions, the survival rates of the CONV and FLASH groups were 64% and 45%, respectively, within 30 days after irradiation. Although the survival rate was higher in the CONV group than in the FLASH group, this difference did not reach statistical significance (Fig. 2d). The average body weight of the mice in both groups declined after irradiation, reaching a minimum average loss of approximately 17% of their initial body weight occurred on day 4 in the conventional group and on day 5 in the FLASH group, followed by gradually recovered (Fig. 2e); however, only a few mice regained their pre-irradiation body weight within 30 days after irradiation. The trend of stool pellet formation paralleled that of body weight, with both groups reaching their lowest counts on day 4. The convention group showed near-complete recovery by day 6, while the FLASH group approached pre-irradiation levels on day 7 (Fig. 2f).

The histological results revealed that the number of regenerating crypts at 84 h after irradiation was comparable between the FLASH and CONV groups (Fig. 2g and h). The degree of intestinal tissue damage was comparable between the FLASH and CONV groups, with a significant reduction in the number of regenerating crypts, extensive destruction of the mucosal layer, and a marked decline in tissue integrity. Severe tissue necrosis and cellular apoptosis were observed in some areas, highlighting the profound impact of radiation-induced damage on intestinal structures. No significant differences in crypt preservation were observed between the two groups, further confirming the similarity between these irradiation modes in this aspect.

### Comparison of FLASH and CONV irradiated mice under high-LET (90 keV/µm)

**Fig. 3:**
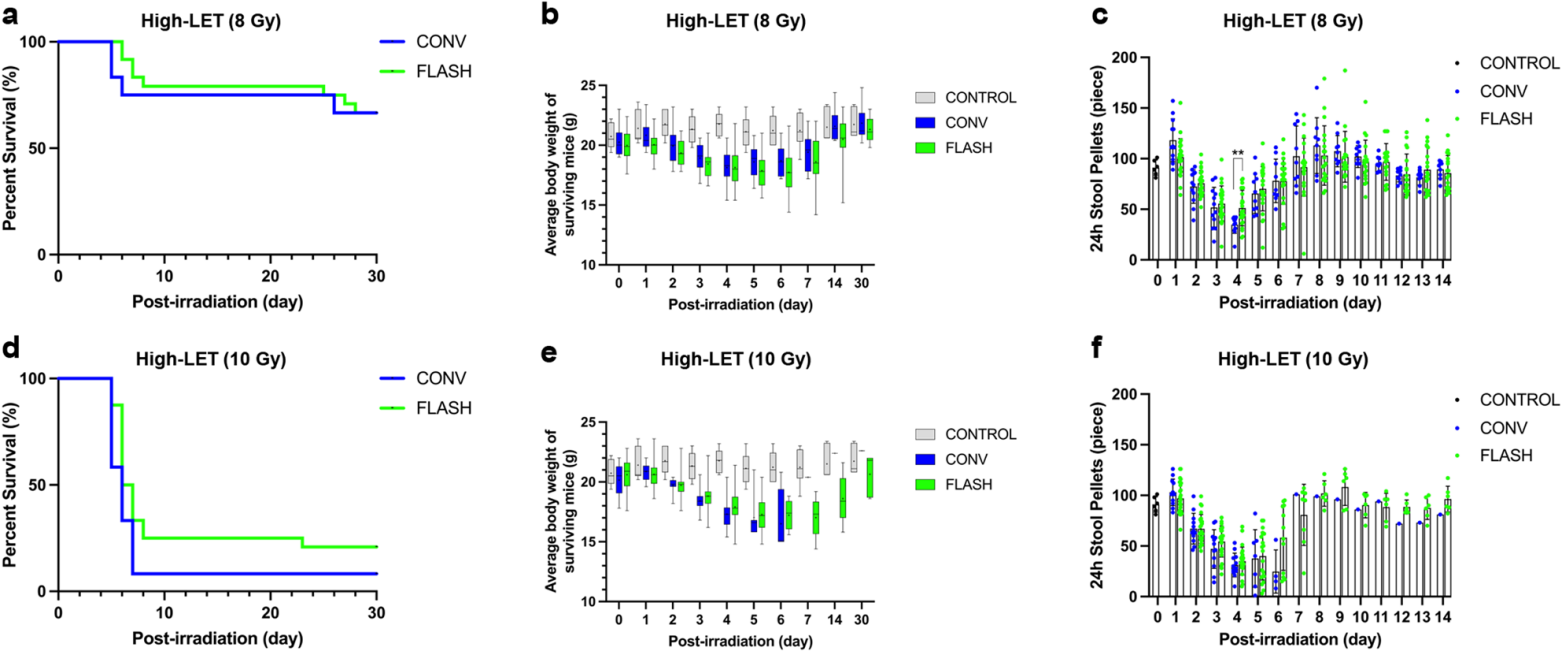
Comparison of FLASH and CONV irradiations under high-LET (90 keV/µm) conditions. (**a**) Survival rate curve of mice that received 8 Gy FLASH or CONV abdominal irradiation in 30 days. (**b**) Daily body weight changes of surviving mice 30 days after irradiation. Each point represents the mean body weight (g) ± standard deviation recorded for all mice in each group at specific time points. (**c**) Quantification of the number of formed stool pellets excreted by unirradiated control mice and mice irradiated with 8 Gy over 24 h at specified time points. (** by the unpaired *t*-test: *p* < 0.05) (**d**) Survival rate curve of mice that received 10 Gy FLASH or CONV abdominal irradiation in 30 days. (**e**) The daily body weight changes of surviving mice 30 days after irradiation. Each point represents the mean body weight (g) ± standard deviation recorded for all mice in each group at specific time points. (**f**) Quantification of the number of formed stool pellets excreted by unirradiated control mice and mice irradiated with 10 Gy over 24 h at specified time points. (**a**–**f**) *n* = 6 in the control group, *n* = 12 in the CONV group and *n* = 24 in the FLASH group

After 30 days of observation at 8 Gy under high-LET conditions, the FLASH and CONV groups had the same survival rate of 67% (Fig. 3a). Although both body weight and stool pellet output showed similar patterns of decline and recovery to their original levels in the two group, the FLASH group exhibited a more gradual and less pronounced changes in both aspects (Fig. 3b and c). The CONV group reached its lowest body weight on day 4, with a 10% reduction from pre-irradiation level, whereas the FLASH group exhibited a more delayed nadir on day 6, with and average loss of 11% (Fig. 3b). By day 14 after irradiation, nearly all mice had regained their original body weight (Fig. 3b). Despite both groups exhibited their minimum average stool pellet formation on day 4, the FLASH group maintained significantly higher output compared to the CONV group (approximately 1.5-fold; *p* = 0.0040) (Fig. 3c).

At 10 Gy under high-LET conditions, the FLASH group had a higher survival rate (20%) than the CONV group (8%) (Fig. 3d). Body weight in both groups declined and subsequently recovered, showing a similar overall trend to that observed at 8 Gy. Notably, the FLASH group exhibited a more gradual decline and a delayed nadir on day 7, compared to day 6 in the CONV group. The maximum average weight loss was 18%, slightly lower than the 16% observed in the CONV group, although recovery in the FLASH group was slower (Fig. 3e). Stool pellet output began to decline in both groups on day 2 after irradiation. However, throughout the entire period, the FLASH group consistently exhibited higher daily pellet counts than the CONV group, with a less pronounced reduction and a more rapid recovery (Fig. 3f).

### Comparison of dose rates above and below 100 Gy/s under high-LET (90 keV/µm) at 10 Gy

**Fig. 4:**
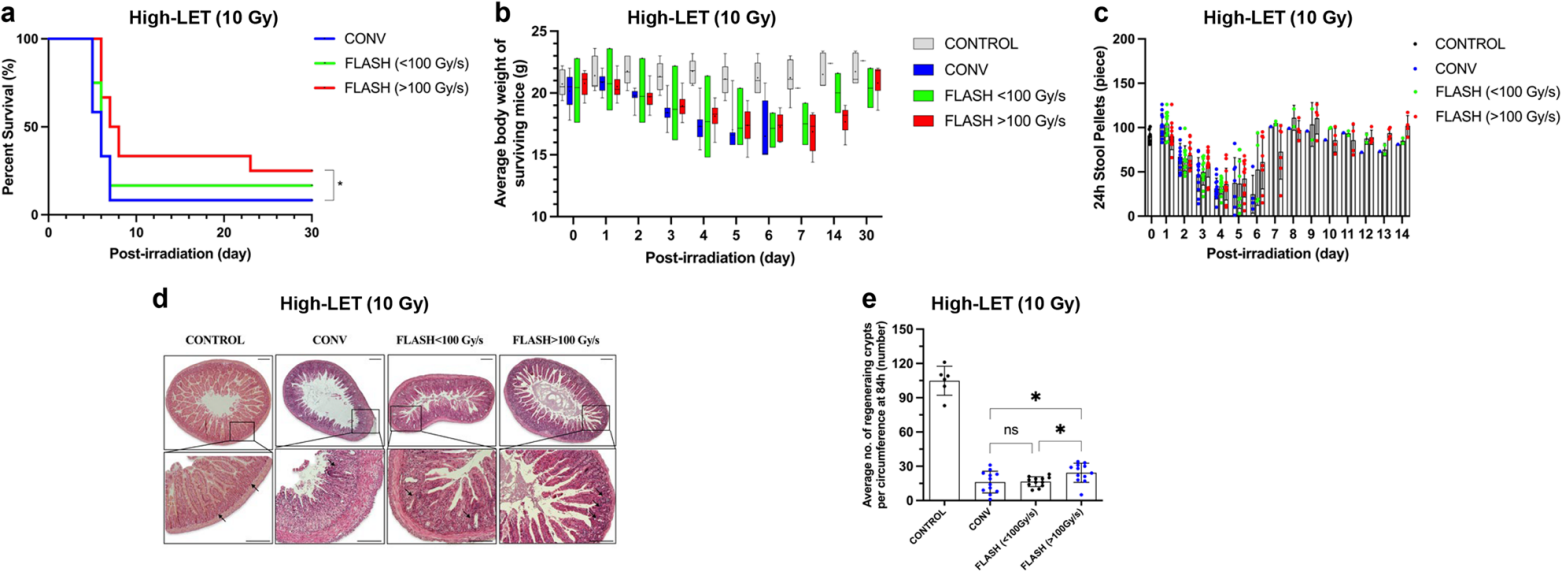
Comparison of dose rates above and below 100 Gy/s under high-LET (90 keV/µm) at 10 Gy. (**a**) Survival rate curve of mice that received 10 Gy FLASH > 100 or < 100 Gy/s and CONV abdominal irradiation in 30 days. (* by long-rank (Mantel–Cox) test: *p* < 0.05) (**b**) Daily body weight changes of surviving mice 30 days after irradiation. Each point represents the mean body weight (g) ± standard deviation recorded for all mice in each group at specific time points. (**c**) Quantification of the number of formed stool pellets excreted by unirradiated control mice and mice irradiated with 10 Gy over 24 h at specified time points. (**d**) Representative hematoxylin and eosin (H&E)-stained cross-sections of the mouse small intestine 84 h after irradiation in the control, CONV, FLASH (> 100 Gy/s), and FLASH (< 100 Gy/s) groups after 10 Gy irradiation. The upper panels show low-magnification views of intestinal morphology; the lower panels present magnified images of the boxed regions. Arrows indicate regenerating intestinal crypts observed 84 h after irradiation. scale bar 100 μm (**e**) Quantification of the average number of regenerating crypts after irradiation. (*P* value = 0.0365) (* by ordinary one-way analysis of variance); (**a**–**c**) *n* = 6 in the control group, *n* = 12 in the CONV group, *n* = 12 in the FLASH (> 100 Gy/s) group, and *n* = 12 in the FLASH (< 100 Gy/s) group. (**d**–**e**) *n* = 4 in the CONV, FLASH (> 100 Gy/s), and FLASH (< 100 Gy/s) groups

The FLASH group exhibited a higher survival rate than the CONV group at 10 Gy; therefore, we considered that it may have a FLASH effect on the intestines under this condition. Considering this finding, we further investigated the relationship between dose rate and FLASH effect on mouse intestines in detail. We divided the FLASH group into two subgroups with different dose rates of >100 Gy/s and <100 Gy/s and compared them with the CONV group. At 30 days after irradiation, the survival rates of the FLASH (> 100 Gy/s), FLASH (< 100 Gy/s), and CONV groups were 25%, 17%, and 8%, respectively. The survival rate was significantly higher in the FLASH (> 100 Gy/s) group compared to the CONV group. The median survival was 7.5 and 6 days, respectively, with a statistically significant difference (*p* = 0.0348) (Fig. 4a). Among all irradiated groups, the FLASH (>100 Gy/s) group exhibited relatively better preservation of body weight and intestinal function, although the differences did not reach statistical significance. As shown in (Fig. 4b), this group experienced a slower decline in body weight during the first 7 days after irradiation and achieved complete recovery by day 30, albeit with a slightly slower recovery trajectory compared to the other two groups. Similarly, stool pellet output in the FLASH (>100 Gy/s) group showed a more stable pattern throughout the observation period. Notably, during the nadir on days 4–5, pellet output remained higher than in the other irradiated groups and gradually returned to pre-irradiation levels thereafter (Fig. 4c).

This observation was further supported by histological analysis, which showed that the number of regenerating crypts in the FLASH (> 100 Gy/s) group was approximately 1.5 times greater than in the CONV group (Fig. 4d and e). The FLASH group also exhibited significantly better preservation of the villus structure, whereas the CONV group showed severe intestinal damage, including notably shortened villi, disrupted architecture, and, in some areas, complete villus loss. In contrast, the villi in the FLASH group more closely resembled those of the control group in terms of length and orderly arrangement. Furthermore, the overall intestinal tissue in the FLASH group remained relatively intact, with better preservation of crypts and mucosal layers and minimal structural damage compared with CONV irradiation.

## Discussion

This study explored the biological impact of carbon-ion FLASH irradiation on the abdominal region of a mouse model. To the best of our knowledge, this was the first report to evaluate the effects of carbon-ion FLASH irradiation on normal tissues under both low- and high-LET conditions, and to further investigate dose rate-dependent FLASH effects using the same abdominal radiation model in detail.

We found that carbon-ion FLASH irradiation delivered at dose rates exceeding 100 Gy/s under high-LET conditions significantly reduced mortality and mitigated acute intestinal damage compared with CONV irradiation, demonstrating a clear FLASH effect. However, the FLASH effect was not observed when the dose rate was below 100 Gy/s under the same high-LET conditions. These findings suggest that the dose rate is an important parameter that influences the FLASH effect. Montay-Gruel et al. reported that the neuroprotective effect of FLASH radiotherapy with electron beams disappears below 30 Gy/s and is fully preserved at dose rates above 100 Gy/s [30], which is consistent with our results.

The FLASH effect was not observed under low-LET conditions. Exposure to 16 Gy low-LET irradiation was associated with reduced survival. This trend could reflect that the total dose exceeded the optimal window for eliciting a protective FLASH effect. Montay- Gruel et al. reported that the magnitude of normal tissue sparing diminishes when the total dose surpasses a certain threshold [36], which is support our results. In contrast, high-LET carbon-ion FLASH irradiation exerted a significant sparing effect on normal tissues, and this finding was further validated through the analysis of regenerating intestinal crypts. Collectively, these findings highlight that both dose rate and LET are critical factors in achieving the FLASH effect with carbon-ions. During the preparation of this manuscript, another group released a report of carbon-ion FLASH experiments using cell lines [26]. In their study, the authors evaluated survival fractions at 19 and 50 keV/µm carbon-ion FLASH irradiation using normal human cells. They found that 50 keV/µm exerted a greater protective effect than 19 keV/µm, further suggesting that LET plays a significant role in modulating the FLASH effect. These findings are consistent with our results and reinforce the importance of optimizing both LET and dose rate for maximizing therapeutic benefit.

From the perspective of track structure and radical formation [37], low-LET radiation produces sparsely distributed ionization tracks that generate diffusible radicals capable of moving through the cell and reacting nonspecifically with DNA. In contrast, high-LET radiation forms densely clustered tracks, where radicals are spatially confined and tend to recombine, resulting in fewer diffusible radicals and reduced indirect chemical damage to DNA. Several studies have shown that during FLASH irradiation, the extremely high instantaneous dose rate rapidly depletes oxygen in the irradiated region, creating a transient hypoxic state. This limits radical oxygen interactions and decreases the formation of oxygen dependent species such as peroxyl radicals. Consequently, total ROS production and oxidative stress are reduced, leading to less biological injury in normal tissues [38, 39]. Taken together, at sufficiently high dose rates, the dense ionization associated with high LET and the transient oxygen depletion induced by FLASH may act synergistically to produce a normal tissue sparing effect, which could partly explain the protective response observed in this study.

A strong correlation was observed between changes in body weight and fecal output in mice following irradiation. Except for the 8 Gy high-LET group, where intestinal function recovered more quickly, both the FLASH and CONV heavy-ion irradiation groups exhibited similar levels of acute intestinal damage. At 10 Gy with dose rates >100 Gy/s, recovery appeared faster in the CONV group; however, this was attributable to survivor bias, as only one mouse survived beyond day 7 and its weight was minimal change post-irradiation. In contrast, three mice in six survived in the FLASH group, showing an initial decline followed by gradual recovery, which resulted in a slower average trend. Thus, considering the enhanced preservation of intestinal stem cells and the functional advantages observed after FLASH, the apparent difference is more likely a consequence of small sample size rather than a true biological disadvantage of FLASH. Although no significant differences in the overall trend of body weight changes were observed between the FLASH and CONV groups, the FLASH >100 Gy/s group exhibited relatively smaller fluctuations in body weight, suggesting a potential sparing effect. Mice with a body weight reduction exceeding 20% had a significantly higher risk of mortality. Before death, mice exhibited marked weight loss and decreased fecal output, and both groups exhibited mortality within 10 days after irradiation. Many deceased mice also presented with abdominal distension. Necropsy revealed intestinal edema caused by intestinal obstruction or inflammation. Potential reasons include severe damage to crypt stem cells induced by irradiation, leading to impaired mucosal repair and nutrient absorption dysfunction; increased intestinal permeability resulting from intestinal injury; and acute inflammatory responses triggering multi-organ failure [40, 41], it appears to be an early effect of radiation. Some mice died 20 days after irradiation. The main reasons may be hematopoietic syndrome. However, the specific causes of death were not further investigated and require detailed examination in future studies.

A primary limitation of radiotherapy is the unintended damage to healthy tissues adjacent to the malignant tumor. Although carbon-ions offer exceptional physical precision, a considerable portion of normal tissue near the planning target volume may still be exposed to high-LET radiation. Our findings demonstrating the tissue-sparing effects of high-LET carbon-ion FLASH irradiation on normal tissues, combined with the fact that carbon-ion therapy is designed to target tumors within the high-LET region, support the clinical potential and feasibility of implementing carbon-ion FLASH therapy.

Although our study employed single-fraction abdominal irradiation, which differs from clinical fractionated regimens, recent reports have demonstrated that intestinal FLASH irradiation can also confer protection under limited multi-fraction schedules [42]. The persistence and magnitude of the FLASH effect across fractionated schemes, however, remain to be fully elucidated and should be addressed in future investigations. We acknowledge that the direct clinical translation of these findings remains limited, and our future work will aim to progressively bridge preclinical research with clinical application.

## Conclusion

Our study revealed that carbon-ion FLASH irradiation delivered at dose rates exceeding 100 Gy/s under high-LET conditions produced a clear tissue-sparing effect, as evidenced by both histological and functional assessments of intestinal tissue. These findings confirm that both dose rate and LET are key factors influencing the biological outcomes of carbon-ion FLASH irradiation. However, further research is needed to uncover the underlying mechanisms driving this protective effect and to explore the role of additional parameters that may influence the efficacy and safety of carbon-ion FLASH therapy.

## Supplementary Information

Below is the link to the electronic supplementary material.

Supplementary Material 1

## Data Availability

The datasets used and/or analysed during the current study are available from the corresponding author on reasonable request.

## Abbreviations

CONV: Conventional dose rate
LET: Linear energy transfer
SOBP: Spread-out Bragg peak
LETd: Dose-averaged linear energy transfer
