# Supplementary material for: Partial abdominal carbon-ion FLASH irradiation spares lethality compared to conventional irradiation: impact of LET and dose rate
Source: Radiat Oncol. 2025 Nov 28;20:186. doi: 10.1186/s13014-025-02765-x (PMC12699875; doi:10.1186/s13014-025-02765-x)
Supplement: Supplementary file 1 — Supplementary Material 1 [file 13014_2025_2765_MOESM1_ESM.pdf]

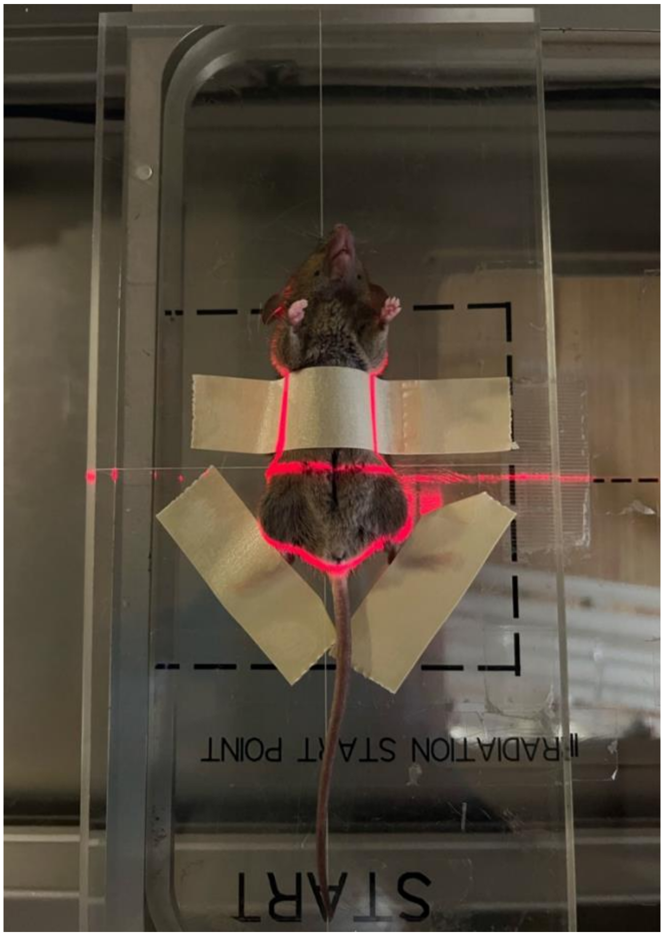

(Supplementary Figure 1) Supine positioning of mice on an acrylic plate for carbon-ion irradiation. A vertical carbon-ion pencil beam was directed at the abdominal region. A red laser guidance system was used to align the mouse so that the abdominal region was positioned precisely at the irradiation isocenter.
